# Comparative evaluation of nerve repair and local tissue response following ReFeel^®^ nerve cuff implantation in a rat sciatic model

**DOI:** 10.3389/fbioe.2026.1759129

**Published:** 2026-04-23

**Authors:** Thomas A. Russell, Antzela Tzagiollari, Bronagh O. Doherty, Orla Burke, Sean Duffy, Paul Dorrell, Masaki Nakamura, Nozomu Watanabe, Gerard M. Insley

**Affiliations:** 1 PBC Biomed, Accelerating Medical Innovation, Limerick, Clare, Ireland; 2 Biomimetic Innovations Ltd (affiliate of PBC Biomed), Limerick, Clare, Ireland; 3 Mochida Pharmaceutical Co Ltd, Tokyo, Japan; 4 Uppsala University, Disciplinary Domain of Science and Technology, Technology, Department of Materials Science and Engineering, Applied Material Science, Uppsala, Sweden

**Keywords:** alginate, collagen, conduit, nerve regeneration, nerve adhesion

## Abstract

**Introduction:**

Peripheral nerve injuries (PNIs) pose a persistent clinical challenge, with current repair strategies limited by material biocompatibility, degradation, and regenerative efficacy. This study evaluates the in vivo performance of ReFeel®, a novel bioresorbable alginate–polyglycolic acid (PGA) nerve cuff, in two rat sciatic nerve models, compared to commercial collagen-based repair devices NeuroMatrix® (conduit) and NeuroMend® (wrap).

**Methods:**

Two studies were conducted in Sprague–Dawley rats using either a 10 mm sciatic nerve gap model (ReFeel® vs. NeuroMatrix®) or a no-gap intact sciatic nerve perineural wrap model (nerve exposed but not transected), comparing ReFeel® with NeuroMend®. Functional, histological, immunohistochemical, and morphometric assessments were performed at 1 week, 8 weeks, and 26 weeks (gap model) and 1 week, 8 weeks, and 13 weeks (wrap model). Tissues were analyzed using hematoxylin and eosin (H&E), Masson’s trichrome, Safranin-O, NF200, S-100, and toluidine blue staining. Degradation, host response, axon regeneration, Schwann cell activity, and fibrosis were quantified.

**Results:**

ReFeel® demonstrated superior Schwann cell alignment and axonal regeneration, particularly at 26 weeks, with near-complete scaffold resorption and minimal fibrotic encapsulation. Quantitative scores showed higher axon densities and comparable G-ratios versus NeuroMatrix®. In the wrap model, ReFeel® matched NeuroMend® in axon count and morphology at 13 weeks, with greater consistency in tissue remodeling. ReFeel® showed earlier material clearance, reduced chronic inflammation, and increased fatty infiltration, suggesting improved biocompatibility and scaffold integration.

**Discussion:**

ReFeel® supports robust peripheral nerve regeneration and degrades in synchrony with tissue remodeling. Compared to collagen-based devices, it enhances central and distal regeneration, promotes restoration of normal nerve homeostasis, and reduces fibrosis and chronic host response. Its ease of application further supports its potential as an effective scaffold for peripheral nerve repair. These findings validate the regenerative potential of alginate–PGA biomaterials and support the clinical translation of ReFeel® as a next-generation nerve repair solution, particularly for bridging nerve gaps or preventing perineural adhesions.

## 1 Introduction

Peripheral nerve injuries (PNIs) present a significant clinical challenge, with an estimated incidence of 16.9 per 100,000 annually in the United States (John et al., 2022). These injuries often result in motor and sensory dysfunction, contributing to long-term morbidity and reduced quality of life (Sumarwoto et al., 2021). Although peripheral nerves possess intrinsic regenerative capacity, outcomes remain suboptimal, influenced by injury severity, anatomical site, and patient-specific factors such as age and comorbidities (Roganovic and Petkovic, 2004).

Surgical intervention remains the mainstay for restoring nerve continuity and guiding axonal regeneration. Direct end-to-end repair is optimal for clean transections with minimal gap and tension (Lin and Jain, 2023). When direct coaptation is not feasible due to tissue loss or tension, alternatives such as nerve conduits or autologous grafts are used. Conduits are most effective for ≤3 cm gaps, offering a protected environment for axonal growth while limiting scar formation (Griffin et al., 2013). Autologous grafts, although biologically favorable, are limited by donor-site morbidity and availability, while allografts often require immunosuppression (Grinsell and Keating, 2014). These limitations underscore the need for bioengineered materials that promote regeneration and minimize adverse responses.

Alginate, a polysaccharide derived from brown algae, has attracted interest for its biocompatibility, biodegradability, and suitability for wound healing, drug delivery, and nerve repair (Ahmad Raus et al., 2021; Zhang et al., 2021; Eisenberg and Hustedt, 2024). Preclinical studies have shown that alginate-based constructs can reduce fibrosis, enhance axon growth, and support local delivery of neurotrophic factors (Zhong et al., 2020; Orive et al., 2006; Breger et al., 2009). Composite materials such as polyglycolic acid (PGA)–alginate hybrids further improve degradation kinetics and reduce inflammation *in vivo* (Hagiwara et al., 2018).

ReFeel® (Mochida Pharmaceutical Co., Ltd.) is an alginate-based nerve cuff composed of a sodium alginate sheet and a nonwoven PGA scaffold. It is designed to reduce perineural fibrosis and support axonal regeneration by providing a biocompatible, bioresorbable structure. This study investigates the *in vivo* performance of ReFeel® in a rat sciatic nerve model, evaluating its degradation profile, host response, and regenerative efficacy. ReFeel® was benchmarked against two collagen-based reference devices: NeuroMend®, a bovine collagen nerve wrap (Masear et al., 2011), and NeuroMatrix®, a collagen-based nerve conduit (Kehoe et al., 2012), using histological, immunohistochemical, and morphometric endpoints. This study aims to generate preclinical evidence supporting the clinical translation of alginate–PGA materials for peripheral nerve repair.

## 2 Materials and methods

### 2.1 Test and reference articles

All test and reference devices (ReFeel®, NeuroMatrix®, and NeuroMend®) were supplied by the respective manufacturers as terminally sterilized medical devices. Sterility was assured by the manufacturers’ validated sterilization processes and verified through accompanying certificates of analysis. Devices were stored under ambient conditions (15–30 °C) until use.

All device preparation and handling prior to implantation were performed under aseptic conditions using sterile instruments within a designated surgical preparation area. No resterilization or additional antimicrobial treatment was applied to any device prior to implantation.

**FIGURE 1:**
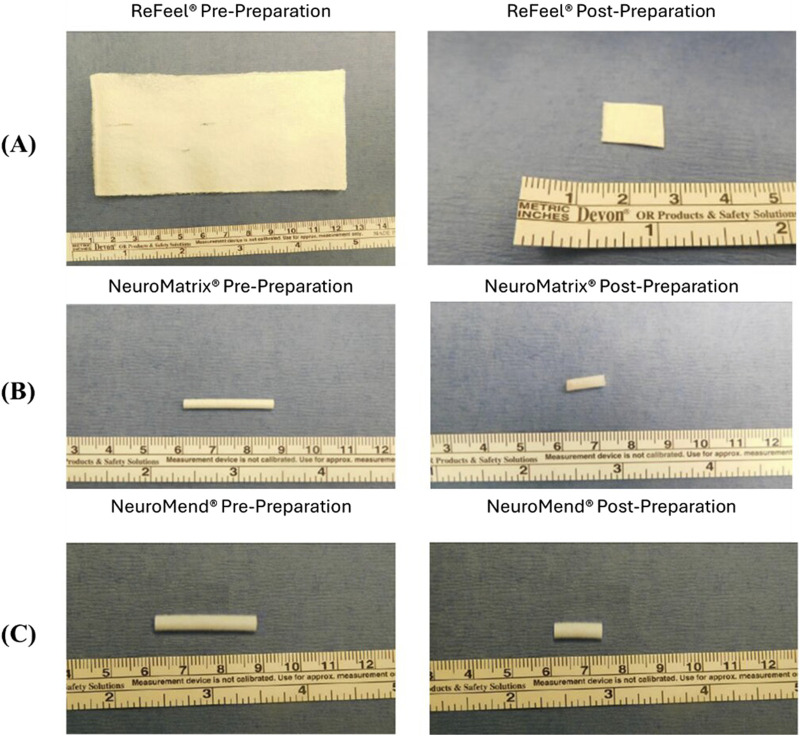
Study devices before and after preparation for implantation: **(A)** ReFeel® nerve cuff pre-preparation and post-preparation following trimming to the target implant dimensions and **(B)** NeuroMatrix® and **(C)** NeuroMend® pre-preparation and post-preparation following cutting to the target length. A metric ruler is included for scale.

ReFeel® is a lyophilized nerve cuff composed of sodium alginate and a PGA mesh, provided in sheets (55–110 mm × 55 mm × 0.3 mm). For implantation, it was cut into ∼10 mm × 12 mm pieces and implanted dry around the sciatic nerve (Figure 1A), with minimal sterile saline added only when required to facilitate handling. Designed for tension-free repair, ReFeel® remained stable under ambient conditions (15 °C–30 °C). Reference devices included Stryker NeuroMend® (bovine collagen wrap, ID: 4.0 mm, L: 25 mm) and NeuroMatrix® (collagen conduit, ID: 2.0–2.5 mm, L: 25 mm) (Figures 1B,C). Both were hydrated in saline for ∼5 min and trimmed to 12 mm before implantation (Figures 1B,C). During placement of NeuroMend®, additional trimming was performed only if required to maintain ∼25% overlap.

### 2.2 Animal model

Two *in vivo* studies were conducted using male Sprague–Dawley rats (Hilltop Lab Animals, PA). In the first study (nerve gap model for ReFeel® vs. NeuroMatrix®), 60 rats were assigned to three groups: ReFeel® (n = 24 implanted), NeuroMatrix® (n = 24 implanted), and Surgical sham (n = 12). Animals were euthanized at pre-specified terminal timepoints of 1 week, 8 weeks, and 26 weeks post-surgery. Per interval, seven implanted animals per article group and four sham animals were euthanized at 1 week and 8 weeks, while all remaining animals were euthanized at 26 weeks (up to 10 implanted animals/group and up to four sham animals at 26 weeks). In the second study (no-gap intact sciatic nerve perineural wrap model; ReFeel® vs. NeuroMend®), 58 rats were assigned to ReFeel® (test; n = 23 implanted), NeuroMend® (sponsor-provided control n = 23 implanted), and surgical sham (n = 12). Animals were euthanized at 1 week, 8 weeks, and 13 weeks post-surgery, with seven implanted animals per article group and four sham animals euthanized at 1 week and 8 weeks, and all remaining animals euthanized at 13 weeks (up to nine implanted animals/group at 13 weeks).

In both studies, animals were monitored with daily general health checks and body weight recording (pre-op, weekly, and pre-terminal). Autotomy/self-mutilation of the treated limb was specifically monitored (scored 0–3), including increased monitoring frequency during the early post-operative period. Neurological assessments (including gait, toe pinch reflex response, posture, and abnormal behavior) were performed pre-operatively, approximately 24 h post-operatively, and then at scheduled intervals through the terminal timepoint (e.g., weekly early on and continuing at defined study weeks through final termination). Due to early terminations, animals could be reassigned between intervals to maintain planned group sizes.

### 2.3 Surgical procedures

In Study 1 (ReFeel® vs. NeuroMatrix®), the left sciatic nerve was exposed via a lateral incision from the proximal thigh to the knee. A ∼12-mm nerve segment was isolated, and an 8-mm section was excised to create a consistent 10-mm gap. In Study 2 (ReFeel® vs. NeuroMend®), the sciatic nerve was exposed through an incision on the left leg beginning at the proximal lateral aspect of the thigh and extending to the level of the knee joint. Exposure without transection was achieved by isolating the sciatic nerve ∼12 mm from surrounding connective tissue midway between the hip and stifle, targeting a consistent ∼10 mm length of nerve exposed, and the wrap/cuff was applied around the intact nerve without excision, while hemostasis was ensured prior to implantation.

**FIGURE 2:**
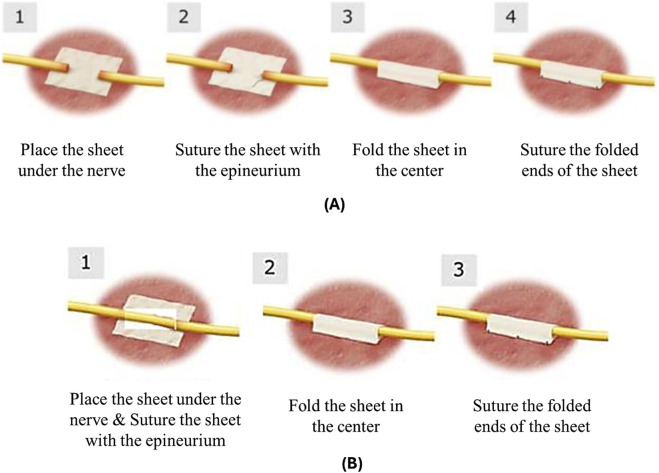
Step-by-step application of ReFeel® in two studies. **(A)** In Study 1, nerve stumps are inserted, and the cuff is closed. **(B)** In Study 2, a similar method is used with slight variation in orientation and closure technique.

**FIGURE 3:**
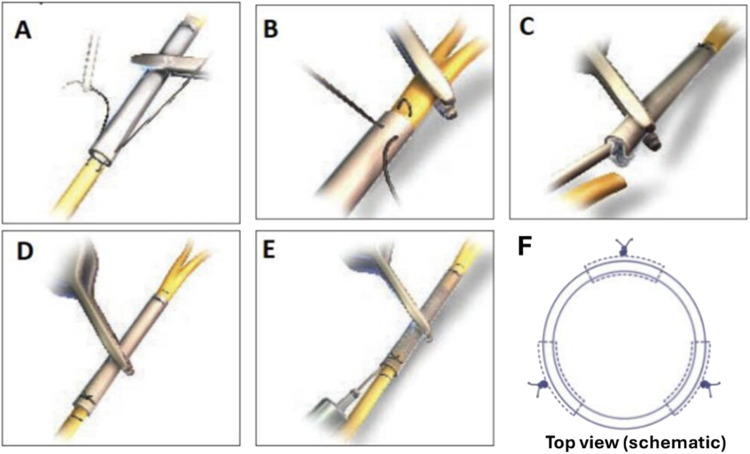
Collagen-device fixation and closure using epineurial sutures (NeuroMatrix® conduit and NeuroMend® wrap). **(A)** A suture is passed through the collagen-device wall near the end/edge. **(B)** A U-shaped epineurial bite is placed longitudinally through the nerve epineurium. **(C)** The suture is returned through the device wall and tied to secure the nerve/device interface. **(D)** The suturing step is repeated to place additional fixation stitches (typically 2–3 per end/edge, as feasible). **(E)** After fixation, the collagen device is aligned/secured over the repair site to stabilize the construct. **(F)** Schematic (top view) illustrating the circumferential closure/overlap and representative suture positions used to secure the collagen wrap/conduit around the nerve.

The ReFeel® sheet was placed beneath the gap, and each nerve stump was sutured to the sheet, folded, and secured (Figures 2A, B, respectively). For NeuroMatrix®, each stump was anchored within the hydrated conduit using 2–3 U-shaped sutures. The epineurial fixation sequence used to secure collagen devices is shown in Figures 3A–D (suture pass through device, longitudinal U-shaped epineurial bite, return pass, and knot). For the NeuroMatrix® conduit (Study 1), these steps were applied at each conduit end following stump insertion to anchor the proximal and distal nerve stumps within the lumen (Figure 3E). The NeuroMend® wrap was hydrated in saline and cut to a target length of ∼12 mm prior to placement. It was wrapped over by ∼25%, and if the overlap exceeded 25%, the excess material was trimmed and secured with interrupted sutures to maintain circumferential closure of the overlapped wrap (schematically shown in Figure 3F). The same suture-handling principle shown in Figures 3A–D (epineurial fixation using atraumatic sutures) underpinned stabilization of both collagen devices, while the device-specific steps differed (stump insertion into a conduit in Study 1 versus wrap overlap/trim and closure in Study 2). Sham animals underwent surgery without material implantation.

### 2.4 Tissue collection and histological evaluation

Tissues collected included left and right sciatic nerves, spinal cord (L3–L5), brain, pelvic bones, and lymph nodes. Following harvest, the spinal column/pelvic tissues with the implant site *in situ* were fixed in 10% neutral buffered formalin and transferred to AnaPath Services GmbH (Liestal, Switzerland) for macroscopic assessment, histological processing, immunohistochemistry, histomorphometry, and histopathological evaluation.

After macroscopic examination and trimming of each implantation site, sciatic nerve samples were paraffin-embedded and processed to obtain defined levels: (i) one cross section proximal to the implant/repair site, (ii) one longitudinal section level proximal to the implant/repair site, (iii) one cross section central to the implant/repair site, and (iv) one cross section distal to the implant/repair site. Paraffin sections were cut at approximately 2–4 μm thickness and stained with hematoxylin and eosin (H&E) and Masson’s trichrome (MT). Sections were additionally evaluated by immunohistochemistry for neurofilament 200 (NF200) to assess axonal regeneration and for S-100 protein (S-100) to assess Schwann cell distribution/activity. Nerve regeneration was then analyzed using a semi-quantitative scoring system for both NF200 and S-100. Regeneration was additionally evaluated using a predefined semi-quantitative 0–4 scoring system applied to immunohistochemistry sections stained for NF200 (axonal regeneration) and S-100 (Schwann cell distribution/activity). Scores were assigned at each predefined section level (proximal, central/device, and distal) based on the extent and continuity of positive staining within the nerve/repair region, where 0 = no detectable signal, 1 = minimal/focal signal, 2 = mild/patchy signal, 3 = moderate/multifocal signal, and 4 = marked/diffuse signal consistent with robust regeneration across the evaluated region. Group-level results represent the mean score per region and marker across evaluable animals at each timepoint.

For assessment of implant/device material status at the implant site, the central cross-section level was additionally stained with Safranin-O and compared against unimplanted control samples processed and stained identically. Where relevant, material detectability/qualitative assessment was supported using stain-dependent visibility and connective tissue staining to aid interpretation of residual material and local tissue remodeling. For quantitative histomorphometry of myelinated fibers, an additional distal cross-section level was processed with osmium tetroxide, embedded in epoxy resin, and stained with toluidine blue (TB). Histomorphometric metrics included myelinated fiber number and density, axon diameter, fiber diameter, g-ratio, myelin thickness, and nerve cross-sectional area. The g-ratio is a dimensionless index of relative myelination calculated as the ratio of axon diameter to total fiber diameter (g-ratio = d_axon_/d_fiber_). Axon diameter was defined as the inner diameter of the myelin sheath, and fiber diameter as the outer diameter, including myelin. Measurements were obtained from osmium-treated, resin-embedded distal cross sections stained with toluidine blue using digital image analysis, and g-ratios were calculated at the individual fiber level and summarized per animal and group.

Whole-slide images were acquired from stained implantation-site sections to support digital review and morphometric quantification. Histomorphometric endpoints were quantified from evaluable sections; therefore, the number of animals contributing to each endpoint may differ from the total enrolled cohort and may vary by timepoint and parameter.

Histopathological evaluation of the implantation site (all sectioning levels) was performed by a qualified pathologist using an adapted/extended ISO 10993-6:2016 (Annex E)-based scoring framework. Microscopic findings were graded on a 0–4 severity scale (0 = within normal limits, 1 = minimal, 2 = mild, 3 = moderate, 4 = marked). Host response parameters included inflammatory cell infiltrates (e.g., polymorphonuclear cells, lymphocytes, plasma cells, macrophages, and multinucleated giant cells), necrosis, neovascularization, fibroplasia/fibrosis, fibrin deposition, edema, hemorrhage, hemosiderin, and mineralization (as applicable), alongside surgery-/nerve-repair-relevant observations, such as adhesions, scar formation/fibrosis, and neuroma formation.

Draining lymph nodes were examined macroscopically and processed for H&E histology, with qualitative/semi-quantitative assessment for evidence of material-related changes and/or tissue response (e.g., histiocytosis/macrophage activity and lymphoid hyperplasia). Contralateral (right) sciatic nerves were processed in selected animals/timepoints for comparison, where specified in the study documentation.

### 2.5 Masking, bias mitigation, and independent assessments

No formal blinding/masking of treatment group was implemented for intraoperative handling and routine post-operative clinical observations (e.g., incision appearance and gait/limb use). To mitigate bias, endpoints and timepoints were pre-specified, assessments followed protocol-defined criteria, and all raw observations (clinical, body weight, neurological assessments, macroscopic findings, and histology/histomorphometry outputs) were recorded contemporaneously and retained in Good Laboratory Practice (GLP) documentation. Histological processing, scanning (whole-slide imaging), histopathology, and quantitative morphometry were performed by an independent third-party pathology laboratory under established protocols, with standardized scoring frameworks and computerized image analysis for morphometric endpoints.

## 3 Results

Results are presented separately for the gap model (ReFeel® vs. NeuroMatrix®) and the wrap/no-gap model (ReFeel® vs. NeuroMend®). In-life observations (body weight, neurological assessments, and clinical findings) are summarized first, followed by macroscopic necropsy findings, and then histopathological/histomorphometric outcomes. Animal-level data supporting in-life observations are provided in Supplementary Tables S1, S2, and data representative macroscopic observations are provided in Supplementary Tables S3–S4.

### 3.1 Surgical and clinical observations

**FIGURE 4:**
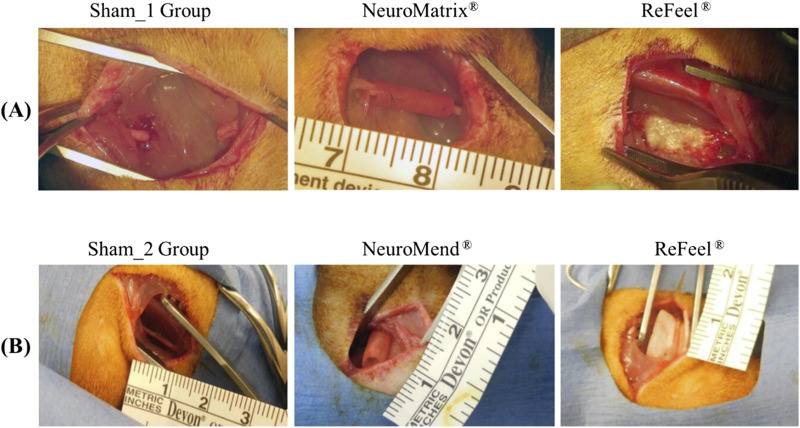
Intraoperative images from the two in vivo studies. **(A)** Study 1: cuff implantation across a 10 mm sciatic nerve gap. **(B)** Study 2: wrap/cuff placement around an intact 10 mm sciatic nerve segment.

All devices were implanted with good conformity to sciatic nerve anatomy (Figure 4). ReFeel® was flexible, easy to handle, either dry or slightly hydrated, and secured with sutures through the epineurium without disruption. NeuroMatrix® and NeuroMend® conduits hydrated quickly and conformed well but required trimming to avoid overlap. All animals were observed with decreased use of the left leg with neurological deficits on the first post-operative day, with most resolving by Day 3. In the ReFeel®–NeuroMatrix® study, post-op limb deficits were resolved by Day 20, while in the ReFeel®–NeuroMend® study, they were resolved by Day 9 (Table 1). Observations associated with the surgical procedure included scabbing/dry lesion and/or alopecia over the incision site. Incidental observations included curling of the digits on the right foot, alopecia on the abdomen, hip, or front legs, or dry scabbing on the face or head. Most animals maintained or gained body weight throughout both studies (Supplementary Tables S1, S2). Body weight remained stable overall, with >10% interim decreases recorded in 1/58 animals (1.7%) in the wrap/no-gap study and 1/60 animals (1.7%) in the gap study (Table 1). All remaining interval-to-interval variations were minor and remained within 10%. No self-mutilation was observed.

**TABLE 1:** Summary of key post-operative clinical observations across the gap (ReFeel^®^ vs. NeuroMatrix^®^ vs. sham) and wrap/no-gap (ReFeel^®^ vs. NeuroMend^®^ vs. sham).

Study model	Total no.	Post-op neurological deficits (Day 1)	Typical resolution of limb-use deficits	Plantar dry lesions (left rear foot)	Clinically relevant weight loss (>10% interim decrease or “weight loss confirmed”)	Self-mutilation/autotomy
Gap model (ReFeel® vs. NeuroMatrix® vs. sham)	60	Common across groups	Generally improved; resolved by ∼ Day 20 (gap study)	23/60 (38.3%); when present, resolved by ∼ Day 20	1/60 (1.7%), “weight loss confirmed”	Not observed
No-gap, intact-nerve wrap model (ReFeel® vs. NeuroMend® vs. sham)	58	Common across groups	Generally improved; resolved by ∼ Day 9 (no-gap study)	Not applicable to this model summary (report as “not assessed/rare”)	1/58 (1.7%) >10% interim decrease	Not observed

Post-operative neurological deficits were common immediately after surgery and generally improved over time. Plantar dry lesions were observed in 23/60 animals (38.3%), resolving by approximately Day 20 when present (Table 1). This is likely due to the neurological deficits and decreased range of motion of the foot. All these observations were associated with the surgical model and were present in all groups at all intervals. In the wrap/no-gap model (ReFeel® vs. NeuroMend®), transient early post-operative deficits were observed with most resolving by Day 3 and all clinically resolved by Day 9 (Table 1). Key non-neurological clinical observations (including collar- and surgery-associated findings) were reported across all groups in the gap study (Table 2) and the wrap/no-gap study (Table 3).

**TABLE 2:** Key non-neurological clinical observations (gap study).

Observation category	(NeuroMatrix®)	Test (ReFeel®)	Sham	Notes/evidence location
Red/dry red substance around the nares/eyes	Reported	Reported	Reported	Report states observed in all animals; commonly associated with Elizabethan collars
Alopecia (often periocular)	Reported	Reported	Reported	Report states alopecia observed in many animals; it may occur after red substance due to grooming
Scabbing around the neck (collar-related)	Reported	Reported	Reported	Report links this to collars
Dry lesions on the plantar surface (left rear foot)	Reported (subset)	Reported (subset)	Reported (subset)	Report states this occurred in some animals, is not unexpected, and resolved by ∼ Day 20 when present; individual animal detail is in Appendix 6
Body weight overall trend	Weight gain overall	Weight gain overall	Weight gain overall	Report states overall weight gain; minor losses generally <10%; individual weights in Appendix 8

**TABLE 3:** Key non-neurological clinical observations (No-gap wrap study).

Observation category	SPC (NeuroMend®)	Test (ReFeel®)	Sham	Notes/evidence location
Post-op decreased use of the left leg/neurological deficits	Reported	Reported	Reported	Report states common on Day 1, mostly resolved by Day 3, rarely beyond Day 3, and no later than Day 9
Red/dry red substance around the nares/eyes	Reported	Reported	Reported	Report states observed in all animals, collar-associated, and may persist briefly
Face swelling/ventral neck or shoulder lesions (collar-related)	Reported (subset)	Reported (subset)	Reported (subset)	Explicitly noted as collar-associated in “clinical observations”
Incision site scabbing/dry lesion and/or alopecia	Reported	Reported	Reported	Listed as surgery-associated observations
Clinically relevant weight loss (>10%)	Not reported	1 animal	Not reported	Report identifies animal 655 (13-week, test) with >10% loss between weeks 1–2, recovered by Day 21
Unscheduled death	—	—	1 animal (sham 673)	Found dead on Day 55; cause undetermined; not related to test article

### 3.2 Macroscopic observations

**FIGURE 5:**
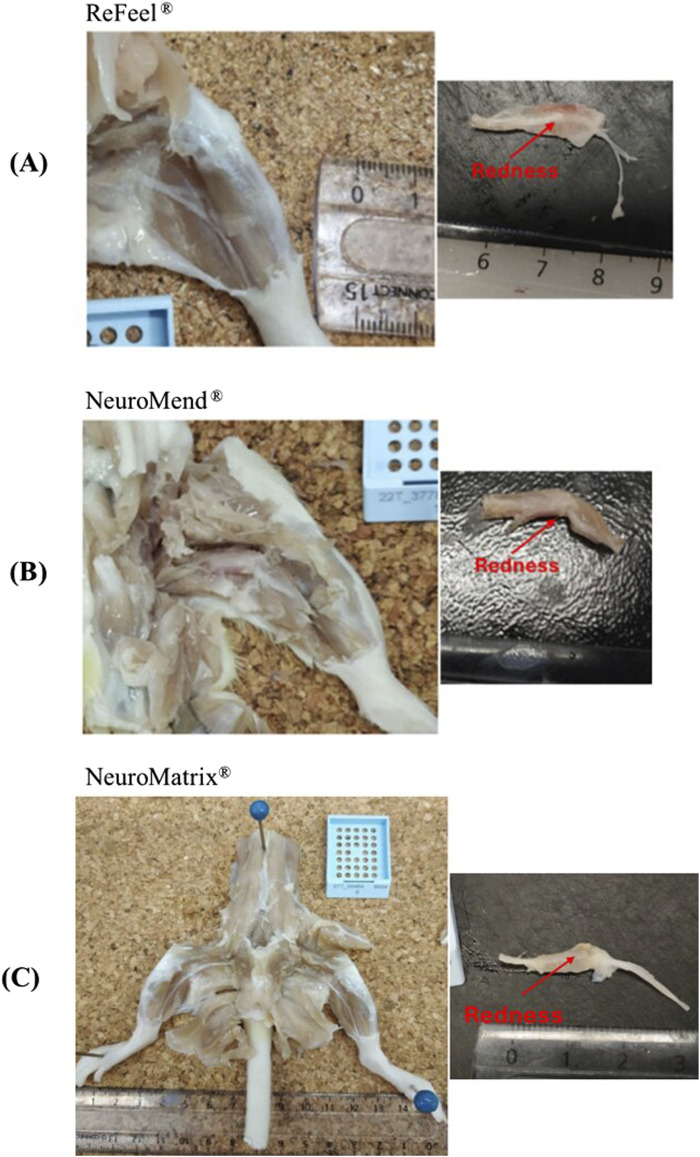
Macroscopic illustrative images at Week 1 of **(A)** the ReFeel®, showing the implantation site, which appeared reddened around the nerve, **(B)** NeuroMend®, and **(C)** NeuroMatrix®, showing the implantation site also appearing reddened, with visible nerve attachment to surrounding tissues.

**FIGURE 6:**
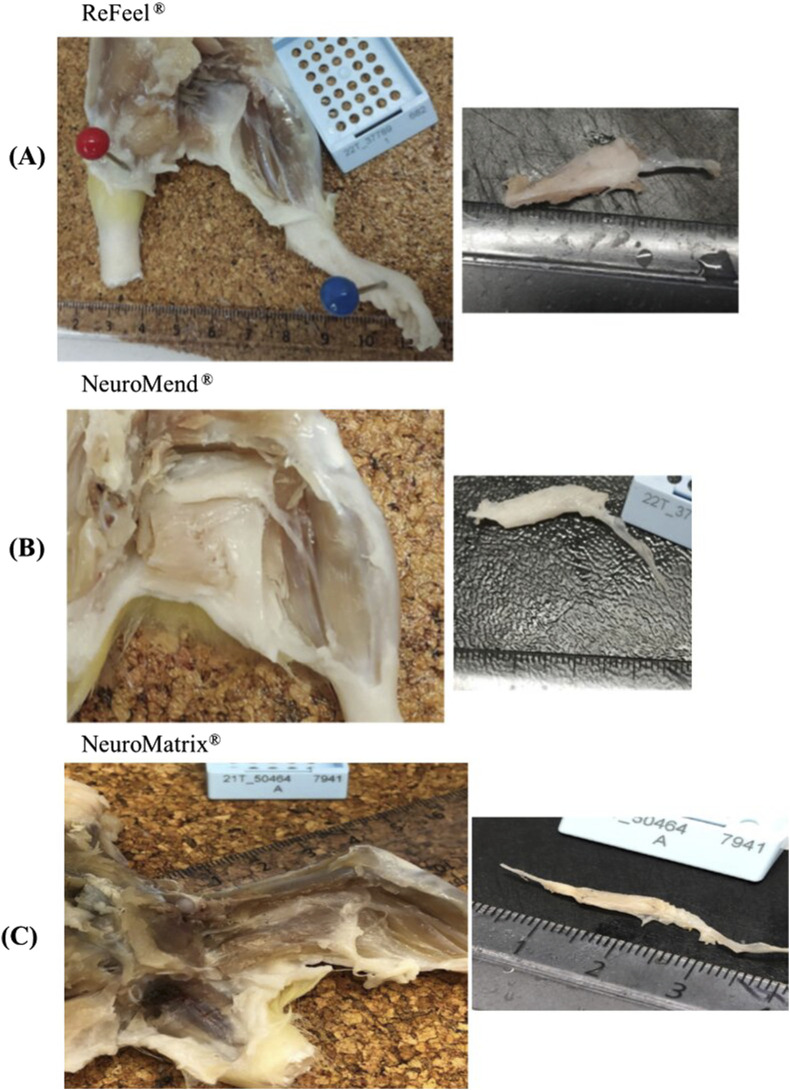
Macroscopic illustrative images at Week 8 of **(A)** ReFeel®, showing partially degraded material, **(B)** NeuroMend®, and **(C)** NeuroMatrix®, showing slight attachment of surrounding tissues.

**FIGURE 7:**
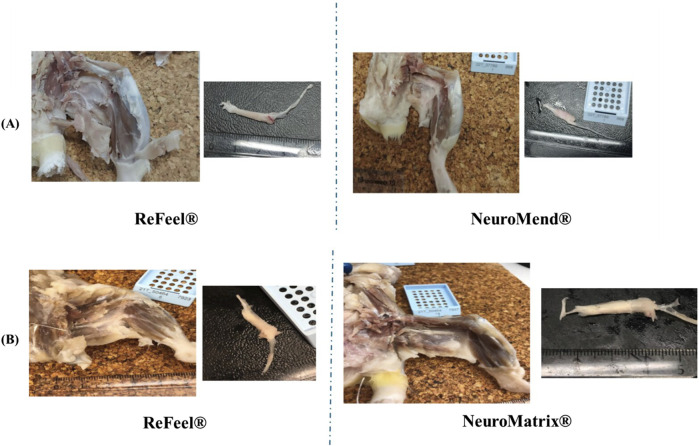
Macroscopic illustrative images at **(A)** Week 13 of ReFeel® and NeuroMend® and **(B)** Week 26 ReFeel®, showing a fully degradable implant and NeuroMatrix®, showing the reddened implantation site and slight fibrosis.

Week 1, ReFeel®, NeuroMend®, and NeuroMatrix® showed evidence of early tissue attachment to surrounding structures consistent with the initial phases of healing. The incidence of implantation-site attachment at Week 1 is summarized in Supplementary Table S3 (gap model) and Supplementary Table S4 (wrap/no-gap model). The ReFeel® group at this interval illustrates the intact state of the implant with slight adhesion to surrounding tissues (Figure 5A). In contrast, NeuroMend® and NeuroMatrix® showed similar adhesion but with slightly more pronounced tissue response (Figures 5B,C). By Week 8, the healing process had progressed significantly, with ReFeel® (Figure 6A) demonstrating reduced gross redness/attachment and a higher incidence of visible device degradation than NeuroMend® and NeuroMatrix® (Figures 6B,C). Macroscopic observations indicated that the ReFeel® sites exhibited minimal adhesions and mild fibrosis, indicating suitable biocompatibility. At 13 weeks, the macroscopic observations highlighted further differences between ReFeel® (Figure 7A) and the NeuroMend® (Figure 7A). The ReFeel® sites showed substantial degradation of the implant, with most samples appearing partially or completely degraded. Findings in the ReFeel®–NeuroMatrix® study at 26 weeks showed complete degradation of the PGA polymer component. Representative Week 26 macroscopic images (gap model) are shown in Figure 7B. A structured incidence summary of macroscopic findings by group and timepoint is provided in Supplementary Table S3 (gap model) and Supplementary Table S4 (wrap/no-gap model).

### 3.3 Histopathological and histomorphometric analyses

**FIGURE 8:**
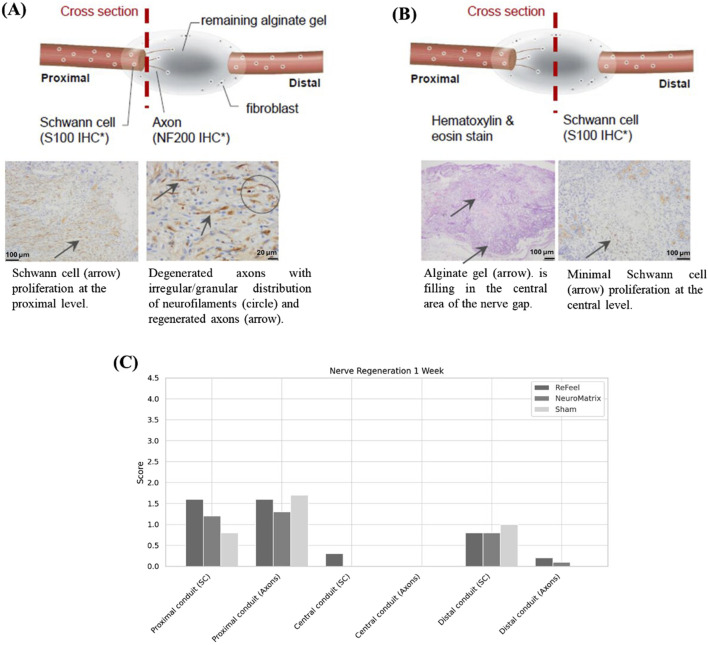
One-week post-implantation. **(A,B)** Greater Schwann cell and axon regeneration in the ReFeel® group, with partial alginate degradation. **(C)** Week-1 semi-quantitative regeneration scores (0–4) by region (proximal/central/distal) for S-100 and NF200 in ReFeel®, NeuroMatrix®, and sham groups. (n = 7/article/interval, n = 4 sham).

At 1-week post-implantation, Schwann cell proliferation was evident at both nerve ends in all groups, with bands of Büngner forming in the proximal segment. ReFeel® began resorbing from the surface inward, facilitating Schwann cell migration and axonal elongation across the scaffold (Figure 8A). Central regions showed limited regeneration overall, but ReFeel® exhibited more aligned Schwann cells and early axon sprouting along its resorbing alginate surface (Figure 8B).

In the gap model, NeuroMatrix® showed limited early Schwann cell alignment and minimal device change at Week 1 (Figure 8C). In the no-gap wrap model, NeuroMend® showed no detectable new nerve formation at Week 1 (semi-quantitative scoring; data not shown). Semi-quantitative scoring demonstrated that ReFeel® supported a stronger early regenerative response than either NeuroMatrix® or sham controls. Proximal Schwann cell (SC) activity was highest in the ReFeel® group (mean score ≈1.5), exceeding NeuroMatrix® (≈1.2) and sham (≈0.6), corresponding to an estimated medium effect size (η^2^ ≈ 0.18) and a trend toward group differences (Kruskal–Wallis, p-value≈0.06). A similar pattern was observed for the proximal axonal (NF200) signal, where ReFeel® (≈1.7) outperformed NeuroMatrix® (≈1.3) and slightly surpassed sham (≈1.5), suggesting earlier axonal sprouting at the repair interface (η^2^ ≈ 0.12; p-value≈0.09). Centrally, all groups showed minimal regeneration, although ReFeel® exhibited a small but detectable SC presence (≈0.2), which was absent in the comparator groups, indicating the earliest signs of scaffold-guided cellular infiltration. Distally, SC activity was comparable across groups, with ReFeel® (≈0.8) exceeding NeuroMatrix® (≈0.6) and approaching sham levels (≈1.0), while ReFeel® was the only group demonstrating a measurable distal NF200 signal (≈0.2). Together, these findings indicate a stronger early Schwann cell and axonal response with ReFeel® compared with NeuroMatrix® and sham at Week 1.

**FIGURE 9:**
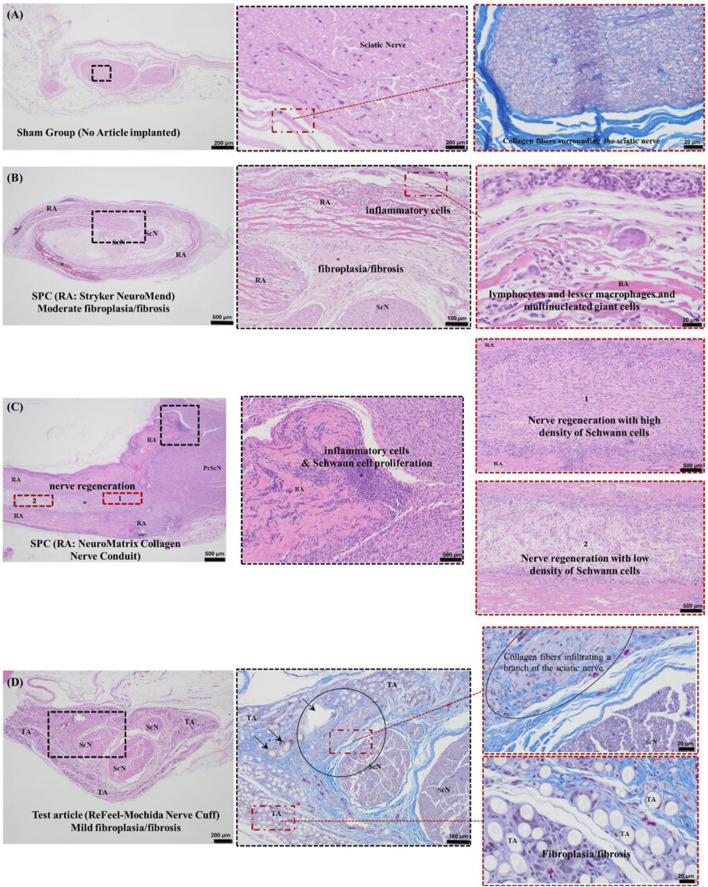
Week-8 histology of the sciatic nerve and implant region (Masson’s trichrome). **(A)** Sham nerve. **(B)** NeuroMend®: peri-implant fibroplasia/fibrosis with inflammatory infiltrate including macrophages and multinucleated giant cells (high magnification). **(C)** NeuroMatrix®: regenerated nerve tissue within the conduit (longitudinal). **(D)** ReFeel®: mild peri-implant fibroplasia/fibrosis with local tissue ingrowth.

**FIGURE 10:**
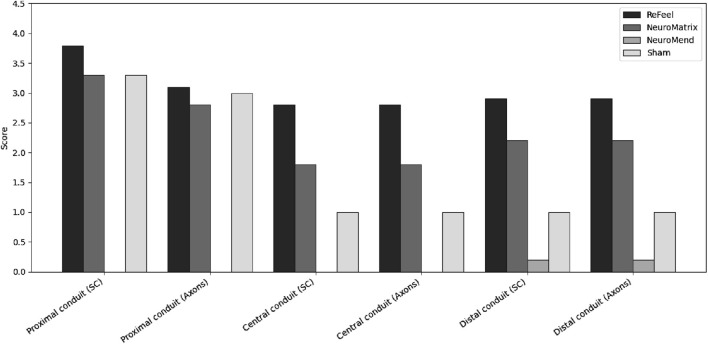
Eight weeks post-implantation. Semi-quantitative nerve regeneration/new nerve formation scores at 8 weeks across the proximal, central, and distal regions. Bars show mean scores (0–4 scale) for ReFeel®, NeuroMatrix®, NeuroMend®, and sham animals. NeuroMend® values derive from the no-gap wrap model, whereas ReFeel®/NeuroMatrix®/sham values derive from the gap model; mean values are shown for contextual comparison and are not from a single head-to-head model. (n = 7/article/interval, n = 4 sham).

**FIGURE 11:**
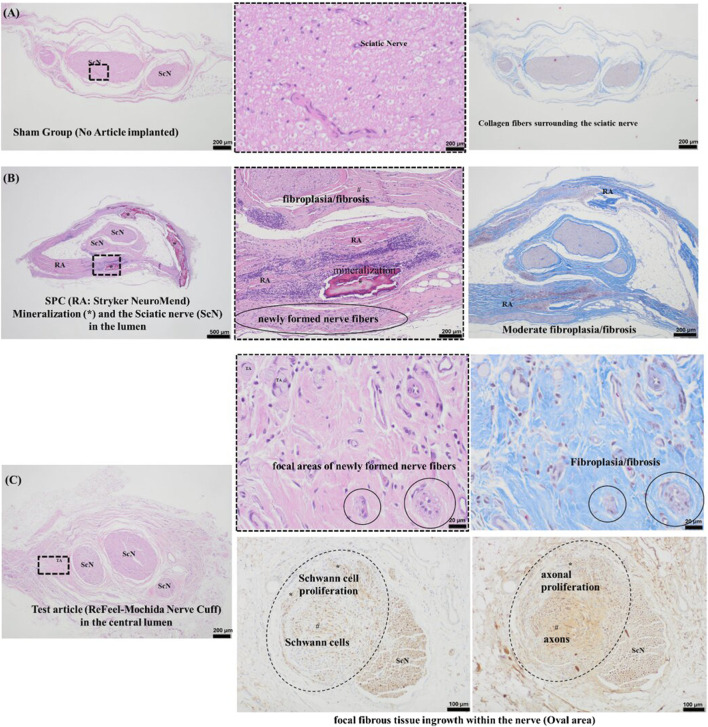
**(A)** Sham. **(B)** NeuroMend® wrap: fibroplasia/fibrosis with focal mineralization (*) and localized new nerve fibers (oval). **(C)** ReFeel®: advanced degradation with peri-implant fibroplasia/fibrosis and focal S-100/NF200 immunoreactivity indicating Schwann cell and axonal presence; in one area, fibrous ingrowth is associated with reduced Schwann cell/axon signal (oval).

At 8 weeks, representative histology is shown for sham (Figure 9A), NeuroMend® (Figure 9B), NeuroMatrix® (Figure 9C), and ReFeel® (Figure 9D). Because Figure 11 includes representative sections from both studies, qualitative comparisons should be interpreted within the relevant model (gap: ReFeel® vs. NeuroMatrix® vs. sham; no-gap wrap: ReFeel® vs. NeuroMend® vs. sham). Across groups, Week-8 histology demonstrated device-associated tissue responses at the implant interface and evidence of nerve tissue organization/regenerative features within the treated region (Figures 9A–D). Semi-quantitative scoring based on S-100 and NF200 immunohistochemistry is summarized in Figure 10.

Host reaction was minimal in the evaluated sham sections (Figure 10A). In implant-contact regions (Section 2: proximal conduit, longitudinal; Section 3: central conduit, cross section; Section 4: distal conduit, cross section), the host response ranged from minimal to moderate in the collagen-device groups and minimal to slight in the ReFeel® group (Figures 10B–D). Macrophage and multinucleated giant cell accumulations associated with suture material were noted, but these were considered procedure-related and were excluded from implant scoring. In the no-gap wrap model, NeuroMend® was associated with inflammatory cell infiltrates surrounding the wrap and fibroplasia/fibrosis within the wrap region, with variable mineralization (Figure 11B). Macrophage involvement is explicitly shown at Week 8 in the NeuroMend® group, where the peri-implant inflammatory infiltrate includes macrophages and multinucleated giant cells (Figure 10B, high-magnification panel). In the gap model, NeuroMatrix® showed peri-implant inflammatory cells and fibroplasia/fibrosis within/around the conduit lumen (Figure 10C). ReFeel® demonstrated visible early device change/PGA scaffold degradation by H&E at Week 8 and exhibited lower overall host-reaction scores versus NeuroMatrix® (average difference −3.2 to −7.6), consistent with a slightly reduced tissue response per ISO 10993–6:2016(E). In the ReFeel® group, implant-contact regions showed a mild inflammatory infiltrate at the device interface (Figure 10D). Semi-quantitative scoring at Week 8 indicated higher Schwann cell and axonal regeneration signals for ReFeel® versus NeuroMatrix® and sham across proximal, central, and distal regions in the gap model (Figure 11). Proximally, Schwann cell activity was highest with ReFeel® (≈3.8) compared with NeuroMatrix® (≈3.3) and sham (≈3.0), with a significant overall group effect (Kruskal–Wallis p ≈ 0.04). Central and distal regions showed a similar pattern, with ReFeel® demonstrating higher Schwann cell and axonal scores than NeuroMatrix® and sham and statistically supported group differences at the central and distal levels (p ≈ 0.02–0.01 and p ≈ 0.03–0.01, respectively; Figure 11). In the no-gap wrap model, regeneration/new nerve formation scores remained low at Week 8 and are shown for contextual comparison (Figure 11), consistent with the expected early remodeling window in an intact-nerve wrap model. Overall, these findings indicate that ReFeel® promotes more consistent and robust regenerative activity throughout the conduit by 8 weeks, outperforming the collagen-based device and untreated controls during this mid-stage healing phase.

By 13 weeks, both NeuroMend® and ReFeel® supported new nerve formation, indicating a shift toward remodeling. Representative histology is shown for sham (Figure 11A), NeuroMend® (Figure 11B), and ReFeel® (Figure 11C). In implant-contact regions, the overall host response was minimal to slight for both NeuroMend® and ReFeel®, and lower in sham animals. Nerve degeneration was minimal and observed in one ReFeel® animal and three NeuroMend® animals, characterized by occasional axonal spheroids and dilation of myelin chambers with intermittent foamy macrophages (Wallerian-type change). No consistent between-group differences in degeneration were observed. Procedure-related inflammatory foci associated with suture material were present in implanted groups and were not attributed to the test or reference device.

**FIGURE 12:**
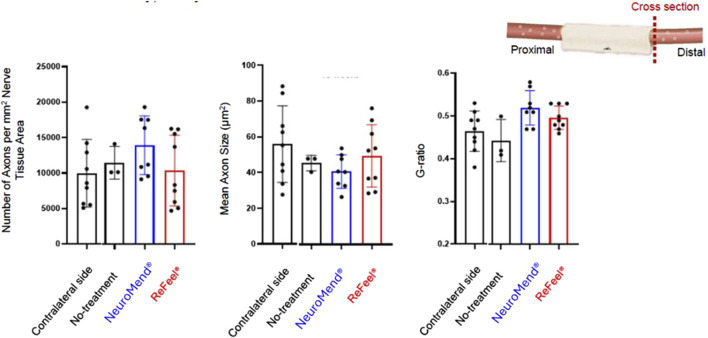
Histomorphometric outcomes at 13 weeks comparing ReFeel® and NeuroMend®. NeuroMend® showed numerically higher axon density and g-ratio, while mean axon size was comparable between groups; differences were not statistically significant (p > 0.05). (n = 9/group). Contralateral = unoperated right sciatic nerve from the same animal; no treatment = operated left nerve in sham animals without device implantation; control = internal processing/analysis control dataset included in the same staining/morphometry run to verify method consistency.

NeuroMend® sections showed fibroplasia/fibrosis within the wrap region and variable mineralization, with focal areas of newly formed nerve fibers adjacent to the device surface (Figure 11B). ReFeel® sections showed advanced material degradation with mild-to-moderate fibroplasia/fibrosis around the implant region and focal areas of Schwann cell (S-100) and axonal (NF200) immunoreactivity consistent with localized regenerative activity; in one ReFeel® animal, fibrous tissue ingrowth into nerve tissue was associated with reduced Schwann cell/axon density (Figure 12C). Macrophages observed in the ReFeel® group resembled those in the NeuroMend® group, suggesting the absence of alginate material at this timepoint. Representative implant-contact inflammatory infiltrates at Week 13 are shown in Figures 11B,C. Minimal to moderate fibroplasia/fibrosis and minimal to slight neovascularization were also observed in the ReFeel® group. Comparative analysis revealed that ReFeel® induced lower average host-reaction scores than NeuroMend®, with differences ranging from −5.72 to −10.33 in sections in contact with the implants. According to ISO 10993–6:2016(E) criteria, these differences represent slight to moderate reductions in host reaction for ReFeel®.

Quantitative histomorphometry at 13 weeks (Figure 12) showed that both ReFeel® and NeuroMend® produced axon density, mean axon size, and g-ratio values within the range of contralateral nerves, with no statistically significant differences between devices (p > 0.05). At 13 weeks in the no-gap wrap model, both ReFeel® and NeuroMend® produced histomorphometric profiles mirroring those of untreated contralateral nerves. NeuroMend showed numerically higher axon density and g-ratio than ReFeel in this dataset; however, differences were not statistically significant (p > 0.05), and both devices produced values within a physiologically appropriate range. ReFeel® showed slightly higher axon numbers and comparable axon morphology, while g-ratio measurements remained within a narrow, physiologically appropriate range for both devices.

**FIGURE 13:**
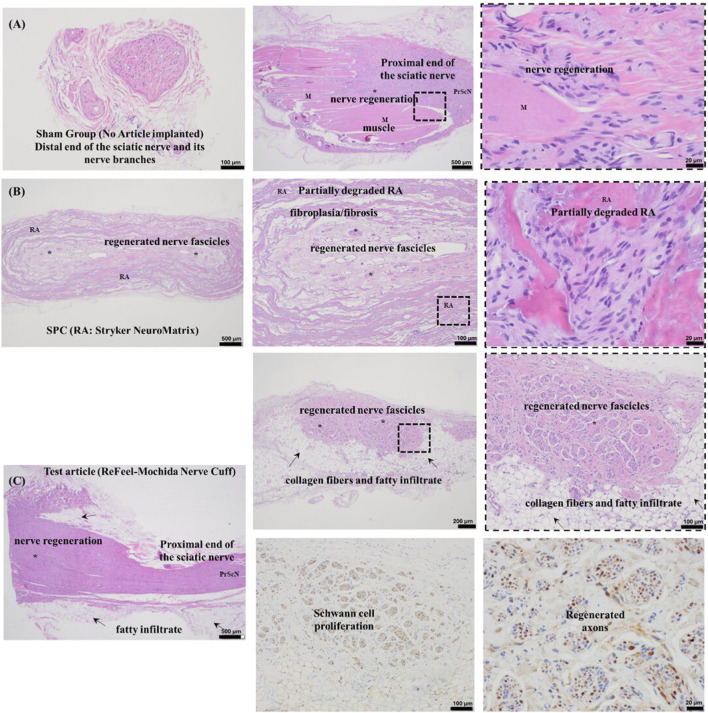
Histological evaluation at Week 26 of nerve regeneration and surrounding structures at the distal end of the sciatic nerve. **(A)** Sham control: Intact morphology with nerve branches and higher magnification showing nerve regeneration in the proximal sciatic nerve and surrounding muscle tissue. **(B)** NeuroMatrix®: Partially degraded scaffold with regenerated nerve fascicles and evident fibroplasia/fibrosis, with higher magnification showing inflammatory and fibrotic tissue surrounding the fascicles. **(C)** ReFeel®: Nerve regeneration with fascicles embedded in collagen fibers and fatty infiltrate. Higher magnifications reveal Schwann cell proliferation and axonal regeneration, demonstrating the regenerative potential of ReFeel®.

Representative Week-26 histology is shown for sham, NeuroMatrix®, and ReFeel® (Figures 13A–C). Sham sections demonstrated intact distal nerve branches and muscle with limited interpretability of the central region because no conduit was present (Figure 13A). In the NeuroMatrix® group, regenerated nerve fascicles were present within the conduit region with associated fibroplasia/fibrosis and partial scaffold disorganization/degradation (Figure 13B). Mild mineralization was noted in some NeuroMatrix® samples, consistent with the histologic appearance of the collagen scaffold and local remodeling at late timepoints (Figure 13B). At Week 26, implant-contact regions showed low-grade inflammatory cell infiltrates in both device groups, with occasional multinucleated giant cells; mild mineralization was observed in some NeuroMatrix® samples (Figures 13B,C). In the ReFeel® group, regenerated nerve fascicles were likewise present, frequently within a remodeled tissue environment that included collagen fibers and fatty infiltrate, with immunohistochemistry confirming Schwann cell and axonal profiles within regenerated fascicles (Figure 13C). Local tissue response in implant-contact regions (proximal/central/distal conduit levels) ranged from slight to moderate in both device groups, with similar overall host-reaction scores. As expected, inflammatory responses adjacent to suture material (macrophages and multinucleated giant cells) were observed and were excluded from device-related scoring. Examples of inflammatory cell infiltrates adjacent to the device region are shown in Figures 13B,C. ReFeel® exhibited moderate to complete degradation by 26 weeks, compared to absent to minimal degradation for NeuroMatrix®. ReFeel® degradation was associated with slightly increased fibroplasia/fibrosis and fatty infiltrate around newly formed nerves compared to NeuroMatrix® and sham control. Comparative analysis indicated similar host reactions between the two articles, with ReFeel® showing slightly lower scores (−3.5 to −0.1), classified as minimal to slight differences per ISO 10993–6:2016(E) criteria.

**FIGURE 14:**
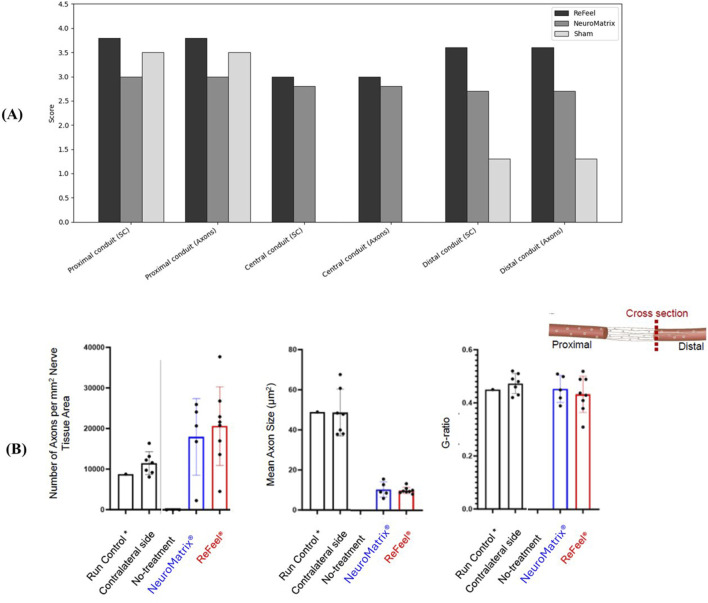
**(A)** Semi-quantitative nerve regeneration scores at 26 weeks in the gap model (ReFeel® vs. NeuroMatrix® vs. sham) at the proximal conduit, central conduit, and distal conduit levels. Central conduit values for sham are shown as 0 because this level was not evaluable in sham animals (no device present). **(B)** Histomorphometric outcomes at 26 weeks in the nerve gap model between ReFeel® and NeuroMatrix® groups. p-value>0.05 (n = 9). contralateral = unoperated right sciatic nerve from the same animal; no treatment = operated left nerve in sham animals without device implantation; run control = internal processing/analysis control dataset included in the same staining/morphometry run to verify method consistency.

At 26 weeks (gap model), both device groups exhibited mature nerve regeneration at the distal level, with ReFeel® showing greater overall regeneration signal by semi-quantitative scoring (Figure 14A). Distal Schwann cell distribution/activity (S-100) and axonal regeneration (NF200) scores remained higher for ReFeel® than NeuroMatrix® and sham, consistent with more extensive regenerated nerve profiles within the conduit region at this timepoint.

Although not statistically significant (p-value>0.05), axon density was consistently higher in ReFeel® at 26 weeks (Figure 14B). Both ReFeel® and the collagen-based comparator devices supported mature nerve regeneration, with no statistically significant differences between groups (p-value>0.05). In the nerve gap model, axon density showed a clear upward trend in the ReFeel® group, which exhibited higher numbers of axons per mm2 than NeuroMatrix®, approaching levels observed in the contralateral uninjured nerve. Mean axon size remained comparable across groups, indicating that regenerating fibers had progressed toward physiological dimensions irrespective of treatment modality. Similarly, g-ratio values are a key indicator of myelin thickness relative to axon caliber, clustered tightly across all groups, with ReFeel® demonstrating values consistent with normal myelination patterns. These findings suggest that by 26 weeks, ReFeel® achieves regeneration quality equivalent to, and in some parameters trending higher than, the collagen conduit. Furthermore, ReFeel® enables stable, well-organized long-term nerve regeneration, while showing trends toward enhanced axonal recovery in some metrics.

**FIGURE 15:**
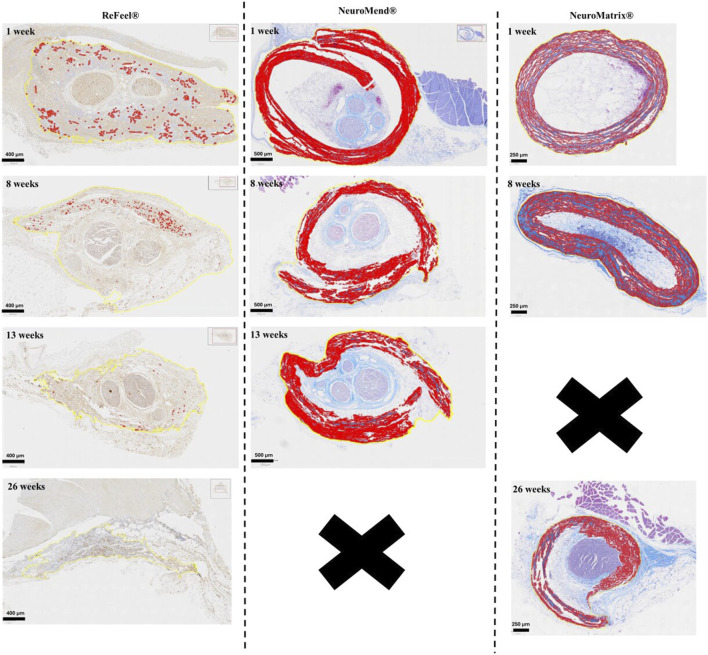
ReFeel® shows rapid resorption and fatty infiltration. NeuroMend® and NeuroMatrix® show persistent collagen with progressive disorganization/partial degradation over time, with NeuroMatrix® demonstrating partial degradation at 26 weeks with residual collagen present.

Because the collagen comparators were evaluated in different in vivo models with different terminal timepoints (NeuroMend® in the no-gap wrap model through Week 13; NeuroMatrix® in the gap model through Week 26), Figure 15 is presented as a within-device time course and should not be interpreted as a direct week-matched comparison across all groups. The ReFeel® degradation profile showed rapid, progressive resorption with foamy macrophages at 1 week, transition to an amorphous residual structure with increased histochemical staining at 8 weeks, and near-complete degradation by 13 weeks (wrap model) and 26 weeks (gap model) (Figure 15). In contrast, the collagen-based comparators demonstrated progressive structural change over time; in the gap model, NeuroMatrix® showed collapse/discontinuity and partial degradation by 26 weeks (loss of collagen architecture), indicating that resorption was underway, although collagen material remained present.

ReFeel®’s resorption coincided with greater fatty infiltration, especially in the distal region, consistent with ongoing tissue remodeling and scaffold integration. Conversely, NeuroMatrix® and NeuroMend® were associated with more prominent peri-implant collagenous organization (e.g., fibroplasia/encapsulation) and inflammatory cells at the implant site at later timepoints. Longitudinal histology indicated that ReFeel® supported regeneration within softer, adipose-rich remodeled tissue, whereas the collagen-based devices frequently showed residual implant collagen at the implant site with a more collagenous peri-implant tissue organization (e.g., encapsulation/adhesions) compared with ReFeel®.

## 4 Discussion

PNIs present a significant clinical challenge due to limited treatment options that consistently restore function. Bioengineered nerve repair devices aim to enhance regeneration while reducing adverse responses, but the ideal scaffold must balance biocompatibility, biodegradability, and support for axonal outgrowth (Faroni et al., 2015). This study evaluated the regenerative capacity and biocompatibility of ReFeel®, an alginate–PGA-based nerve cuff, compared to two collagen-based devices, NeuroMatrix® and NeuroMend®, in rat models of sciatic nerve injury. Across both models, ReFeel® demonstrated regenerative efficacy that was comparable to or exceeded that of the collagen-based reference devices. In the 10 mm nerve gap model (ReFeel® vs. NeuroMatrix®), early axonal regeneration was delayed at 8 weeks in the ReFeel® group relative to NeuroMatrix®, but this trend reversed by 26 weeks, when ReFeel® exhibited greater axonal density and scaffold clearance (Figures 10, 13, 14B, 15). These findings support previous reports that alginate-based constructs can create a permissive environment for axonal elongation and Schwann cell guidance (Ahmad Raus et al., 2021; Zhang et al., 2021; Eisenberg and Hustedt, 2024), achieving mature nerve structure and normalized g-ratios.

In the no-gap intact-nerve perineural wrap model (ReFeel® vs. NeuroMend®), histomorphometry and immunohistochemistry confirmed equivalent axonal regeneration between groups by 13 weeks, with no significant differences in axon count, size, or g-ratio (Figure 12). Because this was a no-gap perineural wrap model (intact nerve), intermediate morphometric differences are more likely to reflect device-specific effects on the local healing environment and perineural mechanical stability (e.g., nerve gliding/adhesion modulation) rather than gap-bridging efficacy. At 13 weeks, NeuroMend® exhibited numerically higher axon density and g-ratio than ReFeel®, although differences were not statistically significant (Figure 12). Both devices produced values within a physiologically appropriate range, supporting comparable remodeling at this timepoint. These results mirror prior work highlighting the capacity of PGA-based scaffolds to promote axonal continuity without impeding regeneration (Kehoe et al., 2012). ReFeel® demonstrated performance that was comparable to, and in some measures trended higher than, collagen-based devices at later study timepoints; however, the durability of these differences requires confirmation in longer-term studies. This is consistent with earlier studies demonstrating that hydrogel-based biomaterials can facilitate nerve repair by mimicking native ECM and reducing scarring (Orive et al., 2006; Breger et al., 2009).

A major differentiator in this study was the degradation behavior of ReFeel®. Importantly, the present study evaluates specific devices rather than material classes in isolation. Accordingly, interpretations emphasize the observed combination of (i) regeneration scoring and morphometry (Figures 10, 12–14A, 14A), (ii) representative histopathology (Figures 9, 11, 14), and (iii) device/material status over time (Figure 15). By 26 weeks, ReFeel® had largely resorbed, whereas the collagen comparators showed slower remodeling with residual collagen still present (Figures 13–15). The progressive material clearance was accompanied by early inflammatory cell infiltration at the implant interface (e.g., macrophage-rich infiltrates visible in the collagen comparator at Week 8 and inflammatory infiltrates at later timepoints), consistent with active remodeling rather than sustained high-grade chronic inflammation (Figures 9B–D). These findings align with literature on the resorbable nature of alginate and PGA and their ability to degrade without inducing persistent foreign body responses (Ahmad Raus et al., 2021; Zhong et al., 2020; Hagiwara et al., 2018). In contrast, the collagen comparators remodeled more slowly and retained residual device material at later timepoints; in the gap model, NeuroMatrix® exhibited partial degradation by 26 weeks, indicating that resorption was underway (Figures 13B, 14B). Importantly, residual collagen-device material does not, by itself, indicate inflammation and should be interpreted in the context of peri-implant organization and the accompanying cellular response. In this rat model, the collagen-based devices more frequently showed a collagenous peri-implant tissue organization (e.g., encapsulation/adhesions) with inflammatory cells present at the implant site (Carnicer-Lombarte et al., 2021; Dubový et al., 2013). These observations should not be generalized to all collagen-based implants, as host response and resorption kinetics are influenced by collagen source, processing/crosslinking, implant architecture, and anatomical site. Complete remodeling may extend beyond the current study duration. While collagen wraps/conduits provided early mechanical support during healing, mechanical performance was not directly assessed in the present study. ReFeel®’s resorption was associated with increased fatty infiltration, especially in distal regions, a marker of tissue remodeling. Fatty tissue and adipose-derived stem cells are known to support nerve healing through paracrine effects and mechanical cushioning (Podsednik et al., 2022; Cherubino et al., 2017; Bucan et al., 2019). The soft tissue integration observed with ReFeel® may facilitate vascularization and perineural restoration. In contrast, the collagen-based devices showed persistent implant collagen at late timepoints (incomplete resorption at 26 weeks) with a more collagenous peri-implant tissue response (i.e., fibroplasia/capsular encapsulation and adhesions) than ReFeel®, which may contribute to reduced nerve gliding and increased perineural tethering. Because the reference devices are collagen-based, residual implant collagen can be difficult to distinguish from collagen-rich host tissue on routine histology. Accordingly, the present interpretation emphasizes peri-implant tissue organization (e.g., encapsulation and adhesions) and the associated inflammatory profile, rather than attributing all collagenous material solely to *de novo* fibrotic deposition.

Host response dynamics differed notably between devices. At 1 week, ReFeel® demonstrated a more prominent macrophage reaction, attributed to early alginate breakdown. However, this resolved by 13–26 weeks, with histological evidence of near-complete degradation and minimal residual fibrosis. Across evaluated sections, ReFeel® was associated with less pronounced collagenous peri-implant organization in representative sections compared with the collagen devices, while both device types exhibited expected post-surgical fibroplasia/fibrosis and inflammatory infiltrates within implant-contact regions (Figures 9, 13). In contrast, NeuroMend® and NeuroMatrix® showed persistent collagen fibers and fibroblastic ingrowth well beyond the expected healing period. These outcomes support the dual role of inflammation in peripheral nerve healing: while necessary for initial regeneration, prolonged activation may lead to scarring and impaired function (Dubový et al., 2013; Benowitz and Popovich, 2011). ReFeel® appears to balance this response effectively by enabling early immune engagement followed by scaffold clearance and tissue remodeling. These findings are consistent with the broader literature showing that host response depends on material chemistry, architecture, and degradation kinetics, and that matching material clearance to the pace of tissue remodeling can help limit prolonged foreign-body–type responses. However, immune responses vary widely by formulation and implant context; therefore, the present observations should be interpreted as device- and model-specific rather than a general statement that alginate universally modulates immune responses more effectively than collagen (Carnicer-Lombarte et al., 2021; Dubový et al., 2013; Benowitz and Popovich, 2011).

While these findings demonstrate the regenerative and immunological benefits of ReFeel®, certain limitations should be acknowledged. First, functional outcomes (e.g., gait analysis and electrophysiology) were not a primary endpoint. A limitation of this work is that objective functional endpoints were not included as primary efficacy measures. The study was designed to focus on GLP histopathology and quantitative morphometry as standardized indicators of nerve regeneration and local tissue response; however, future studies should incorporate dedicated functional testing to directly link structural regeneration with functional recovery. Second, variability in histological scoring and occasional surgical artifacts (e.g., fibrotic ingrowth near suture sites) may have influenced results in isolated animals. Finally, this study evaluated outcomes up to 26 weeks. Longer-term follow-up (and larger animal models) would further clarify late-stage remodeling, including completion of resorption of comparator collagen implants and durability of the regenerated nerve architecture.

## Data Availability

The raw data supporting the conclusions of this article will be made available by the authors, without undue reservation.

## Supplementary material

SUPPLEMENATARY TABLE S1 Body weight summary by study, group, and terminal timepoint.

SUPPLEMENATARY TABLE S2 Mean body weight percentage change from pre-op to terminal by study, group, and timepoint.

SUPPLEMENATARY TABLE S3 Macroscopic findings at the sciatic nerve implantation site (gap model).

SUPPLEMENATARY TABLE S4 Macroscopic findings at the sciatic nerve implantation site (wrap/no-gap model).
