# Supplementary material for: Comparative evaluation of nerve repair and local tissue response following ReFeel® nerve cuff implantation in a rat sciatic model
Source: Front Bioeng Biotechnol. 2026 Apr 23;14:1759129. doi: 10.3389/fbioe.2026.1759129 (PMC13150485; doi:10.3389/fbioe.2026.1759129)
Supplement: Supplementary file 1 [file Table1.docx]

**Table S1. Body weight summary by study, group, and terminal timepoint**

**Table S1.** Body weight (g) summary by study model, treatment group, and terminal timepoint. Values are mean ± SD. “Pre-op” refers to the body weight recorded prior to surgery; “Terminal” refers to the body weight recorded at the scheduled necropsy timepoint.

**Wrap/no-gap model (ReFeel® vs NeuroMend® vs Sham)**

| Terminal timepoint | Group | N | Pre-op (g), mean ± SD | Terminal (g), mean ± SD |
| --- | --- | --- | --- | --- |
| 1 week | ReFeel® | 7 | 252.6 ± 10.3 | 270.9 ± 15.9 |
| 1 week | NeuroMend® | 7 | 248.1 ± 12.2 | 268.4 ± 22.8 |
| 1 week | Sham | 4 | 246.8 ± 17.4 | 278.0 ± 24.8 |
| 8 weeks | ReFeel® | 7 | 245.4 ± 8.0 | 439.0 ± 29.7 |
| 8 weeks | NeuroMend® | 7 | 251.0 ± 12.4 | 420.7 ± 28.0 |
| 8 weeks | Sham | 4 | 251.8 ± 1.7 | 450.8 ± 22.0 |
| 13 weeks | ReFeel® | 9 | 241.2 ± 7.2 | 483.1 ± 26.4 |
| 13 weeks | NeuroMend® | 9 | 236.0 ± 8.7 | 457.0 ± 39.4 |
| 13 weeks | Sham | 3 | 240.3 ± 8.8 | 468.0 ± 55.8 |

**Gap model (ReFeel® vs NeuroMatrix® vs Sham)**

| Terminal timepoint | Group | N (enrolled) | N with terminal weight | Pre-op (g), mean ± SD | Terminal (g), mean ± SD |
| --- | --- | --- | --- | --- | --- |
| 1 week | ReFeel® | 7 | 7 | 281.0 ± 11.7 | 278.0 ± 21.0 |
| 1 week | NeuroMatrix® | 13 | 13 | 278.7 ± 20.3 | 294.8 ± 17.2 |
| 1 week | Sham | 2 | 2 | 283.5 ± 20.5 | 286.5 ± 6.4 |
| 8 weeks | ReFeel® | 8 | 6 | 297.4 ± 19.9 | 393.2 ± 21.3 |
| 8 weeks | NeuroMatrix® | 6 | 5 | 296.7 ± 18.5 | 408.8 ± 33.5 |
| 8 weeks | Sham | 3 | 3 | 301.7 ± 21.9 | 412.7 ± 37.3 |
| 26 weeks | ReFeel® | 10 | 9 | 293.6 ± 21.2 | 523.2 ± 37.2 |
| 26 weeks | NeuroMatrix® | 10 | 10 | 288.8 ± 11.0 | 581.3 ± 47.1 |
| 26 weeks | Sham | 3 | 3 | 315.3 ± 14.0 | 560.7 ± 10.0 |
