# Supplementary material for: Comparative evaluation of nerve repair and local tissue response following ReFeel® nerve cuff implantation in a rat sciatic model
Source: Front Bioeng Biotechnol. 2026 Apr 23;14:1759129. doi: 10.3389/fbioe.2026.1759129 (PMC13150485; doi:10.3389/fbioe.2026.1759129)
Supplement: Supplementary file 2 [file Table2.docx]

**Table S2. Mean body weight percentage change from pre-op to terminal by study, group, and timepoint**

**Table S2.** Mean percentage change in body weight from pre-operative baseline to terminal timepoint by study model, treatment group, and terminal timepoint. Percent change was calculated as: Values are derived from group mean body weights reported in Table Sx.

**Wrap/no-gap model (ReFeel® vs NeuroMend® vs Sham)**

| Terminal timepoint | Group | Pre-op mean (g) | Terminal mean (g) | Mean % change (pre-op → terminal) |
| --- | --- | --- | --- | --- |
| 1 week | ReFeel® | 252.6 | 270.9 | +7.2% |
| 1 week | NeuroMend® | 248.1 | 268.4 | +8.2% |
| 1 week | Sham | 246.8 | 278.0 | +12.6% |
| 8 weeks | ReFeel® | 245.4 | 439.0 | +78.9% |
| 8 weeks | NeuroMend® | 251.0 | 420.7 | +67.6% |
| 8 weeks | Sham | 251.8 | 450.8 | +79.0% |
| 13 weeks | ReFeel® | 241.2 | 483.1 | +100.3% |
| 13 weeks | NeuroMend® | 236.0 | 457.0 | +93.6% |
| 13 weeks | Sham | 240.3 | 468.0 | +94.8% |

**Gap model (ReFeel® vs NeuroMatrix® vs Sham)**

| Terminal timepoint | Group | Pre-op mean (g) | Terminal mean (g) | Mean % change (pre-op → terminal) |
| --- | --- | --- | --- | --- |
| 1 week | ReFeel® | 281.0 | 278.0 | −1.1% |
| 1 week | NeuroMatrix® | 278.7 | 294.8 | +5.8% |
| 1 week | Sham | 283.5 | 286.5 | +1.1% |
| 8 weeks | ReFeel® | 297.4 | 393.2 | +32.2% |
| 8 weeks | NeuroMatrix® | 296.7 | 408.8 | +37.8% |
| 8 weeks | Sham | 301.7 | 412.7 | +36.8% |
| 26 weeks | ReFeel® | 293.6 | 523.2 | +78.2% |
| 26 weeks | NeuroMatrix® | 288.8 | 581.3 | +101.3% |
| 26 weeks | Sham | 315.3 | 560.7 | +77.8% |

**Notes:**

1. Percent change values are computed from group mean weights.
2. Small negative change at 1 week can occur due to immediate post-operative effects and normal short-term variability.
