# Supplementary material for: Comparative evaluation of nerve repair and local tissue response following ReFeel® nerve cuff implantation in a rat sciatic model
Source: Front Bioeng Biotechnol. 2026 Apr 23;14:1759129. doi: 10.3389/fbioe.2026.1759129 (PMC13150485; doi:10.3389/fbioe.2026.1759129)
Supplement: Supplementary file 3 [file Table3.docx]

**Table S3. Macroscopic findings at the sciatic nerve implantation site (gap model)**

**Table S3.** Incidence of macroscopic findings at the sciatic nerve implantation site in the 10 mm gap model (ReFeel® vs NeuroMatrix® vs Sham) at terminal timepoints (1, 8, and 26 weeks). Values are x/n (affected/assessed). “N/A” indicates the parameter is not applicable to sham animals (no implant present).

| **Finding** | **ReFeel® 1w** | **ReFeel® 8w** | **ReFeel® 26w** | **NeuroMatrix® 1w** | **NeuroMatrix® 8w** | **NeuroMatrix® 26w** | **Sham 1w** | **Sham 8w** | **Sham 26w** |
| --- | --- | --- | --- | --- | --- | --- | --- | --- | --- |
| Implantation site / sciatic nerve: red | 3/7 | 0/8 | 0/8 | 4/8 | 0/6 | 0/6 | 0/4 | 0/3 | 0/3 |
| Implantation site / sciatic nerve: white | 0/7 | 0/8 | 0/8 | 0/8 | 0/6 | 0/6 | 0/4 | 0/3 | 0/3 |
| Implantation site / sciatic nerve: gelatinous | 0/7 | 0/8 | 0/8 | 0/8 | 0/6 | 0/6 | 0/4 | 0/3 | 0/3 |
| Implant swollen | 2/7 | 0/8 | 1/8 | 0/8 | 0/6 | 0/6 | N/A | N/A | N/A |
| Implant degraded | 0/7 | 6/8 | 7/8 | 0/8 | 4/6 | 6/6 | N/A | N/A | N/A |
| Attachment to surrounding tissue | 7/7 | 6/8 | 0/8 | 8/8 | 4/6 | 0/6 | 0/4 | 0/3 | 0/3 |

**Notes:**

1. “Attachment to surrounding tissue” refers to gross adhesion/attachment noted during necropsy trimming of the implantation site.
2. Sham animals underwent surgical exposure without device implantation; therefore implant-specific findings are listed as N/A.
