# Supplementary material for: Comparative evaluation of nerve repair and local tissue response following ReFeel® nerve cuff implantation in a rat sciatic model
Source: Front Bioeng Biotechnol. 2026 Apr 23;14:1759129. doi: 10.3389/fbioe.2026.1759129 (PMC13150485; doi:10.3389/fbioe.2026.1759129)
Supplement: Supplementary file 4 [file Table4.docx]

**Table S4. Macroscopic findings at the sciatic nerve implantation site (wrap/no-gap model)**

**Table S4.** Incidence of macroscopic findings at the sciatic nerve implantation site in the wrap/no-gap (intact-nerve) model (ReFeel® vs NeuroMend® vs Sham) at terminal timepoints (1, 8, and 13 weeks). Values are x/n (affected/assessed). “N/A” indicates the parameter is not applicable to sham animals (no implant present).

| **Finding** | **ReFeel® 1w** | **ReFeel® 8w** | **ReFeel® 13w** | **NeuroMend® 1w** | **NeuroMend® 8w** | **NeuroMend® 13w** | **Sham 1w** | **Sham 8w** | **Sham 13w** |
| --- | --- | --- | --- | --- | --- | --- | --- | --- | --- |
| Implantation site / sciatic nerve: red | 1/7 | 3/7 | 3/9 | 1/7 | 0/7 | 1/9 | 0/4 | 0/4 | 0/3 |
| Implantation site / sciatic nerve: white | 0/7 | 0/7 | 0/9 | 0/7 | 0/7 | 0/9 | 0/4 | 0/4 | 0/3 |
| Implantation site / sciatic nerve: gelatinous | 0/7 | 0/7 | 0/9 | 0/7 | 0/7 | 0/9 | 0/4 | 0/4 | 0/3 |
| Fibrosis at implantation site | 0/7 | 0/7 | 0/9 | 0/7 | 0/7 | 1/9 | 0/4 | 0/4 | 0/3 |
| Implant degraded | 1/7 | 7/7 | 8/9 | 0/7 | 0/7 | 0/9 | N/A | N/A | N/A |
| Attachment to surrounding tissue | 1/7 | 0/7 | 0/9 | 1/7 | 5/7 | 4/9 | 0/4 | 0/4 | 0/3 |

**Notes:**

1. “Wrap/no-gap model” indicates the sciatic nerve was exposed and instrumented without transection (no nerve segment excised).
2. Sham animals underwent surgical exposure without device implantation; therefore implant-specific findings are listed as N/A.
